# Respiratory Diseases

**Published:** 1912-05

**Authors:** 


### Respiratory Diseases.

Respiratory diseases are necessarily catarrhal in their character, for the respiratory passages are lined with mucous epithelium whose function it is to secrete mucus, and a disease of these passages naturally manifests itself in an altered secretion of mucus, both as to quality and quantity. It is quite likely that such changes in the functionation of the mucous membranes might, to a limited degree, be brought about by sheerly vaso-motor or mechanical influences, by a deficient or excessive blood supply, or by mechanical irritation, and it is quite certain that vaso-motor and mechanical influences *are* important factors in the production of all disorders of the respiratory tract; however, it is nowadays pretty generally recognized that in the severer types of catarrh which attack the respiratory passages these internal factors are not sufficient to explain or to account for the phenomena; that, in fact, they play only a predisposing rôle, in that they lower the resisting power of the epithelial tissue, and that the active morbid agent is in every case a pathogenic germ which finds lodgment upon the weakened membrane.

The frequency with which the upper respiratory passages are victimized by catarrhal processes has, no doubt, something to do with the exposure of these parts to the changes of temperature and barometer, and to the irritating dust and smoke that are drawn into the nose and throat in the act of breathing, all of which tend to impair both the blood supply and the texture of the epithelium, and render it an easy prey to infection, but it depends chiefly upon the fact that this same exposure to the atmosphere lays the upper respiratory passages peculiarly open to invasion by pathogenic bacteria, and offers an inviting point for their entrance into the system. Indeed, so well is this truth established that we know the naso-pharyngeal membranes to be the constant seat of many micro-organisms, which lie in wait, so to speak, for the least sign of weakening on the part of the membrane to drive home their attack. In the ordinary types of catarrh it is not always the same germ that is responsible for the trouble; usually it is a mixed infection. Only in certain well-marked diseases is one specific germ predominant, as in diphtheria and pneumonia, but we have come to recognize that in all cases of catarrh the essential etiology is the same, that of a bacterial infection grafted on a tissue of lowered resistance, differing only in the virulence of the germ and the area attacked.

The general course of these respiratory catarrhs is naturally the same. They exhibit three general stages: (1) The stage of invasion, and of course of resistance, too, characterized by a more or less violent systemic and local disturbance manifested by fever, pain, congestion, and suspension of secretion; (2) the stage of exudation, marking the termination of the battle royal between the invaders and the body defenses, and characterized by the reactionary relaxation of the tissues and profusion of mucu-purulent secretions; and (3) the stage of resolution, in which the circulatory mechanism begins the task of clearing away the debris and reconstructing the injured tissues.

In every one of these stages, therefore, there are two prime and distinct indications for therapeutic interference. First, the defenses of the body must be aided by inhibiting, as much as possible, the toxicity of those germs which have gained an entrance and begun to proliferate, and by preventing the entrance of others, to which the air passages, as already noted, are peculiarly liable. Second, the scavenger forces of the body must be assisted by thoroughly cleansing and disinfecting the wreckage, and the recuperative processes stimulated by healing and soothing agents. All of which must be done without further, and perhaps worse, injury to the tissues by the agent employed.

From these premises it has come to be generally recognized by physicians that in the respiratory diseases Listerine has its largest and most effective field of usefulness. As the respiratory passages are peculiarly susceptible to the invasion of pathogenic bacteria and noxious germs, so also they are peculiarly amenable to the application of local, direct antisepsis. All that is needed to complete the connection between demand and fulfillment is a preparation which shall meet the requirements of the case without injury to the tissues, and this preparation is supplied in Listerine.

During the last decade Listerine has become thoroughly identified by the leaders of thought in medical practice as one of the standard remedies in the treatment of disease. Its universal use, its efficient formula, and its purity and uniformity, have made it practically officinal. Contributors to our best periodicals and authors of our standard text-books mention and advocate it unhesitatingly. The recognition which it has won in conservative England, and on the continent, corroborates the opinion of the American profession regarding Listerine.

